# Three Impossibilities

**Published:** 1862-04

**Authors:** 


**THREE IMPOSSIBILITIES.**—To overestimate the greatness of redeeming love. To overestimate the joys which God hath prepared for those who love him. To overestimate the obligation under which we are laid to consecrate our time, our talents, our fortunes, and all that we have and are, to the promotion of God's glory, and the happiness of our fellow-men. With such a consecration, no man has ever avowed, or ever can say, on a dying-bed, that if he had his life to live over again, he would serve his Maker less zealously, and would do less for his country and his kind.

